# Ultra-fast data sanitization of SRAM by back-biasing to resist a cold boot attack

**DOI:** 10.1038/s41598-021-03994-2

**Published:** 2022-01-07

**Authors:** Seong-Joo Han, Joon-Kyu Han, Gyeong-Jun Yun, Mun-Woo Lee, Ji-Man Yu, Yang-Kyu Choi

**Affiliations:** School of Electrical Engineering, Korea Advanced Institute of Science and Technology, (KAIST) 291 Daehak-ro, Yuseong-gu, Daejeon, 34141 Republic of Korea

**Keywords:** Engineering, Nanoscience and technology

## Abstract

Although SRAM is a well-established type of volatile memory, data remanence has been observed at low temperature even for a power-off state, and thus it is vulnerable to a physical cold boot attack. To address this, an ultra-fast data sanitization method within 5 ns is demonstrated with physics-based simulations for avoidance of the cold boot attack to SRAM. Back-bias, which can control device parameters of CMOS, such as threshold voltage and leakage current, was utilized for the ultra-fast data sanitization. It is applicable to temporary erasing with data recoverability against a low-level attack as well as permanent erasing with data irrecoverability against a high-level attack.

## Introduction

Static random access memory (SRAM), which is a type of volatile memory, is widely used for temporary storage of encryption keys and secret data in security systems^1–3^. It is commonly believed that stored data in SRAM are lost immediately and instantly when power is removed, and this is the main reason why SRAM is considered a secured memory device for high-level security applications. However, data remanence at low temperature was reported in power-off SRAM^4,5^. Recently, studies on a cold boot attack to SRAM have been reported^6,7^. If the time of data remanence is prolonged at low temperature, encryption keys and secret data can be decoded via a cold boot attack by a hacker. A method for fast erasing in SRAM is therefore necessary against the cold boot attack. A few approaches were demonstrated to prevent SRAM data from being decoded by the physical cold boot attack by use of additional circuitry including an erase transistor, storage capacitor, and charge pump. However, they sacrificed layout efficiency and increased hardware cost^8,9^. In addition, long time of 0.2 μs was needed for data erasing.

In this work, an ultra-fast data sanitization of SRAM within 5 ns is demonstrated by use of forward back-biasing against the cold boot attack. Back-bias applied to a body of a metal–oxide–semiconductor field-effect transistor (MOSFET) is utilized to delete stored data via intentional distortion of the latch state between two inverters of a SRAM cell, which also encloses two n-channel pass-gate MOSFETs.

These two inverters are cross-coupled to sustain the latch state stably as long as power is supplied. An inverter is composed of a complementary metal–oxide–semiconductor (CMOS), *i*.*e*., a pull-down n-channel MOSFET abbreviated NMOS and a pull-up p-channel MOSFET abbreviated PMOS. In the proposed data sanitization, two types of data erasing are available. One is temporary erasing by symmetric application of back-bias to two p-channel MOSFETs in each inverter. The other is permanent erasing by asymmetric application of back-bias to a PMOS in one inverter and to an NMOS in the other inverter. In the former case, data recovery is allowed after a low-level threat attempt by hacking. In the latter case, data recovery is impossible after an attempt of a high-level threat by hacking. Temporary erasing partially disturbs data reading by application of the symmetric forward back-bias during an attack and then the partially distorted data are recoverable after the cessation of the hacking attempt. In contrast, permanent erasing completely deletes remnant data by application of the asymmetric forward back-bias against the critical hacking attempt and thereafter the erased data are irrecoverable. Therefore, the user can reuse the previous data with temporal erasing, whereas one cannot do them with permanent erasing when the hacker’s attack is finished. This approach with the aid of the back-biasing does not demand additional circuitry because back-biasing is commonly used for tuning CMOS characteristics, such as threshold voltage (*V*_T_) or leakage current (*I*_OFF_)^10–12^. The data sanitization mechanism is analyzed for both the permanent erasing and the temporary erasing with physics-based device simulations. The results show that the proposed back-bias scheme can provide immunity against a cold boot attack at low temperature.

## Methods

For the simulations of a SRAM cell, MOSFETs with a high-*k* gate dielectric and a metal gate for a 32 nm technology node were modeled with the aid of a SILVACO ATLAS TCAD simulator^13^. The detailed parameters of the NMOS were set by referring to^14^. Thereafter those of the PMOS were regenerated as a counter-part of the NMOS. Based on the device-level simulations, a conventional cell of six transistor-SRAM (6 T-SRAM) was constructed using ATLAS mixed-mode TCAD simulations to confirm the behaviors of the SRAM data sanitization by forward back-biasing. It is well known that the 6 T-SRAM is composed of two pull-up PMOS, two pull-down NMOS, and two pass-gate NMOS.

**Figure 1:**
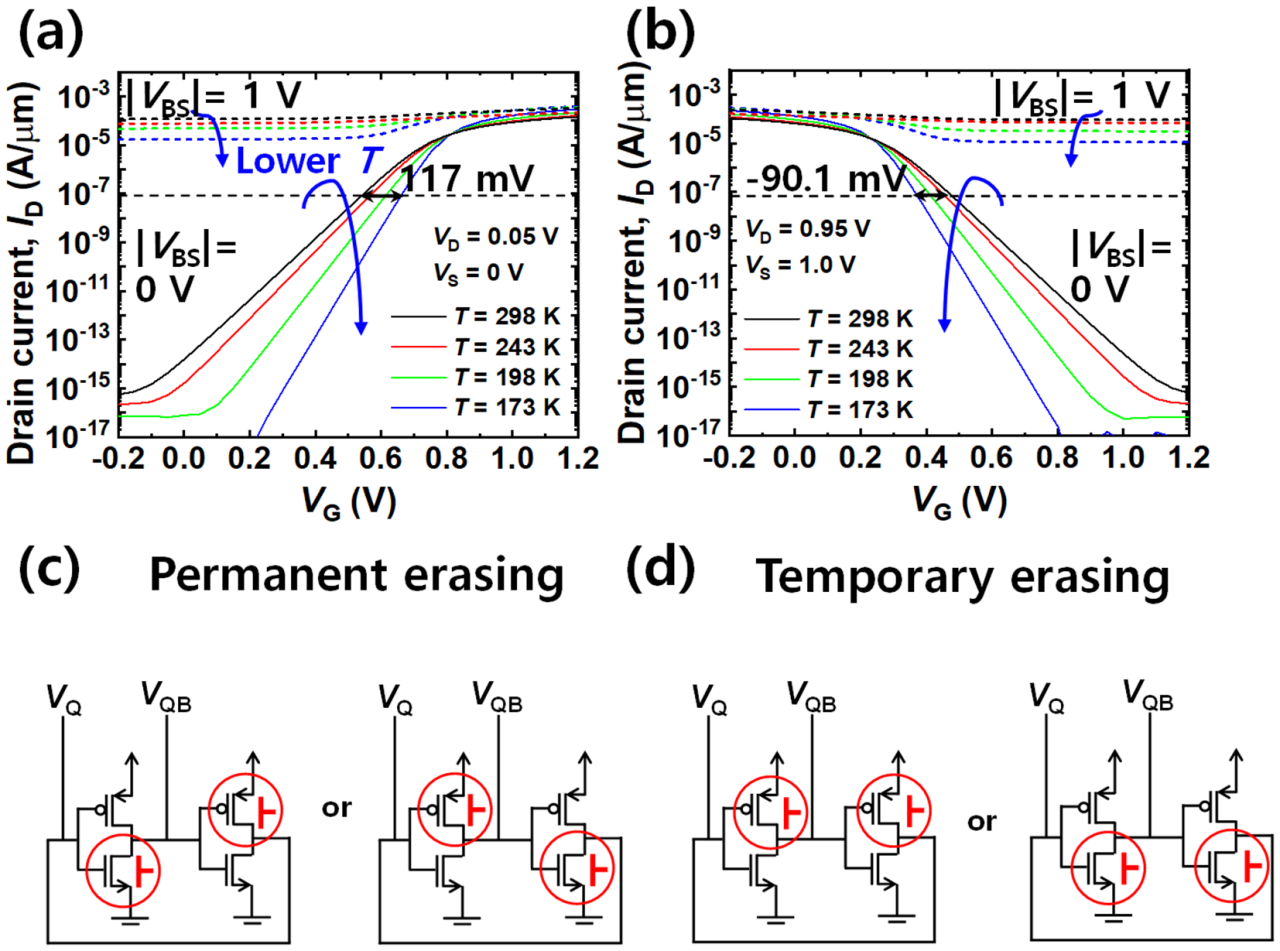
Transfer characteristics (*I*_D_-*V*_G_) and back-bias (|*V*_BS_|) schemes in a SRAM cell. (**a**) Plot of *I*_D_-*V*_G_ in NMOS. (**b**) Plot of *I*_D_-*V*_G_ in PMOS. (**c**) Schematic of asymmetric back-biasing for permanent erasing. (**d**) Schematic of symmetric back-biasing for temporary erasing.

In detail, the gate length (*L*_G_), the gate width (*W*_G_), and the equivalent oxide thickness (*EOT*) of the gate dielectric are 45 nm, 1 μm and 1.53 nm, respectively in the device level simulations with single MOSFET. The doping concentration of the source (*N*_source_), drain (*N*_drain_), and substrate (*N*_sub_) was set as 1 $\times$ 10^20^ cm^−3^, 1 $\times$ 10^20^ cm^−3^, and 3 $\times$ 10^18^ cm^−3^, respectively. The dopant polarity for the PMOS was opposite to that for the NMOS. Various physical models, such as Schockley-Read-Hall (SRH), bandgap narrowing (BGN), Fermi–Dirac (FERMI), non-local band-to-band tunneling (BTBT), trap-assisted tunneling (TAT), and Cryogenic (CRYO) were used for accurate physics-based simulations. As a result, transfer characteristics (*I*_D_-*V*_G_) of the NMOS and PMOS modulated by forward back-bias (*V*_BS_) were obtained, as shown in Fig. 1a and b. Note that the forward *V*_BS_ of the NMOS is 1 V and the forward *V*_BS_ of the PMOS is -1 V. This bias mode is opposite to that of the conventional back-bias scheme that usually relies on a reverse mode. Under the forward back-biasing, both NMOS and PMOS were turned on regardless of *V*_G_. Figure 1a and b also show that the *I*_D_-*V*_G_ characteristics of the NMOS and the PMOS were influenced by temperature (*T*). As *T* is lowered, the subthreshold slope (*SS*) becomes steeper and the off-state current (*I*_OFF_), referred to as leakage current, tends to be decreased. When a cold boot attack was attempted at 173 K, remnant data were read even at a power-off state owing to the improved SS and suppressed *I*_OFF_^6,7^. It is inferred that the cold boot attack can be avoided by intentionally heating up the SRAM far above room temperature when the hacking attempt is sensed. However, it is practically difficult to apply heat to the SRAM. Moreover, this approach is not effective because the shift of *V*_T_ by temperature change, expressed as *dV*_T_/*dT*, is very small. It was found that *dV*_T_/*dT* was 0.94 mV/K for the NMOS and -0.72 mV/K for the PMOS from Fig. 1a and b. These values are comparable to the experimental data reported in^15,16^. As an example, *ΔT* (= *T*_high_ – *T*_300K_) of 426 K is required to make a *ΔV*_T_ of 0.4 V that can distort the *I*_D_-*V*_G_. This means that high temperature (*T*_high_) of 726 K is needed to induce the *ΔV*_T_ of 0.4 V solely by temperature at room temperature. Such high temperature can provoke serious damage to the package of a SRAM chip or a PCB board owing to melting. In contrast, a *ΔV*_T_ of 0.4 V is achievable by the back-bias of below 0.8 V. This reveals that the forward back-biasing is more effective than increment of temperature to avoid the cold boot attack.

Using the modeled CMOS, a SRAM cell was designed to examine the feasibility of data sanitization by forward back- biasing. The behaviors of the SRAM sanitization were verified using a SILVACO ATLAS mixed-mode TCAD simulation. The 6 T-SRAM was composed of two pull-up PMOS, two pull-down NMOS, and two pass-gate NMOS. The word-line (*V*_WL_) voltage of the pass-gate MOSFETs was low for turning them off and isolating the storage node (SN) and bit-lines (BLs). The *W*_G_ of the MOSFET comprising the SRAM cell was set as 80 nm referring to^17^. The supply voltage (*V*_DD_) for SRAM operation was set to 1 V. For permanent erasing, asymmetric forward back-bias was applied to the NMOS of the left inverter and the PMOS of the right inverter or vice versa, as depicted in Fig. 1c. Note that the magnitude of the forward back-bias is the same for the NMOS and the PMOS, whereas they have opposite voltage polarity. In this case, the initial data state can be reset to ‘0’ or ‘1’. For temporary erasing, symmetric forward back-bias was applied to the PMOS of both inverters or the NMOS of both inverters, as depicted in Fig. 1d. In this case, the latch state locked in both inverters can be distorted when initially off-state PMOSs or NMOSs are turned on not by gate bias but by the applied back-bias, as shown in Fig. 1a and b.

## Results

### Permanent erasing

**Figure 2:**
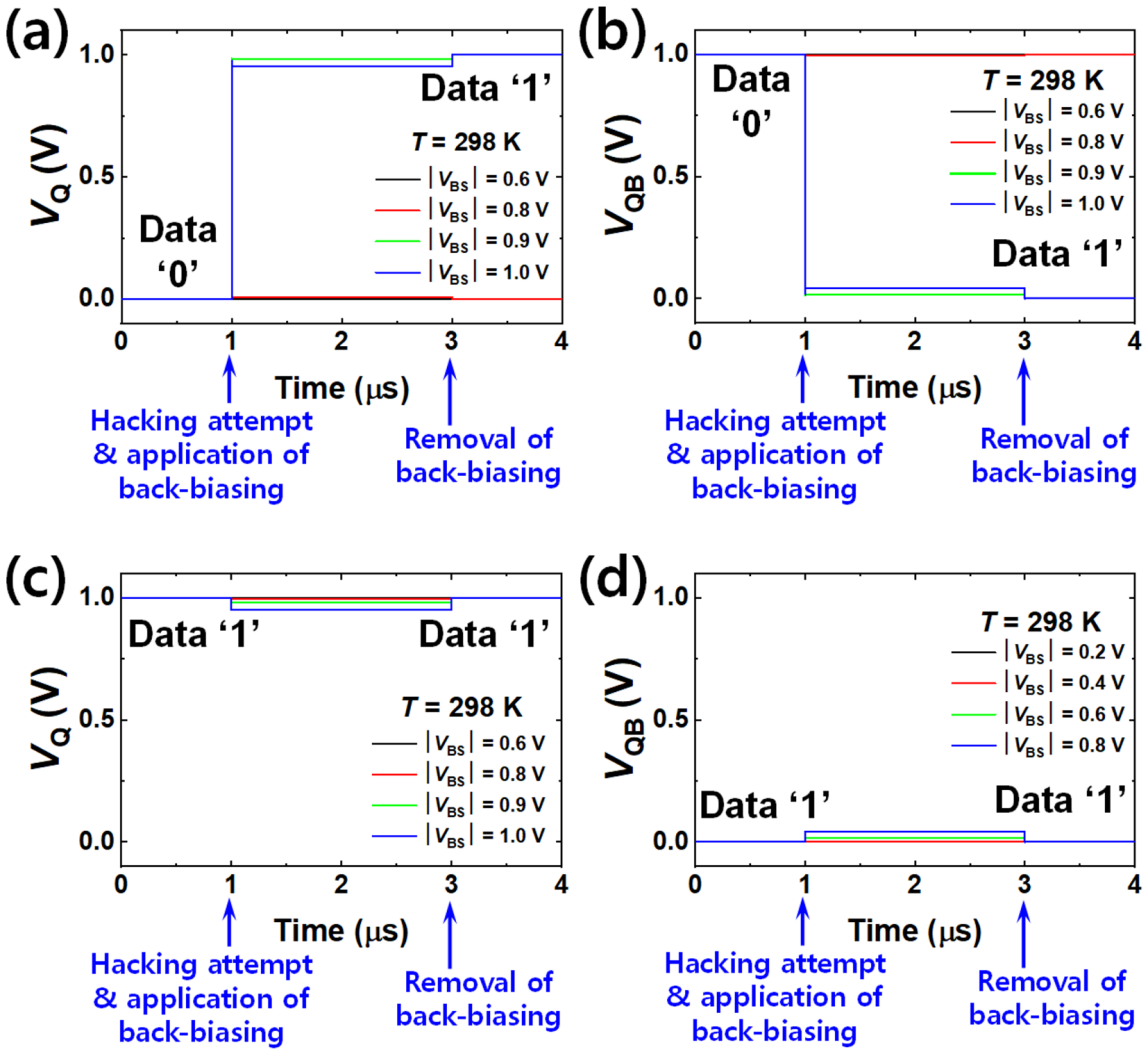
Permanent erasing characteristics at room temperature (298 K). (**a**) Bit line voltage (*V*_Q_) for various |*V*_BS_| with transition from ‘0’ to ‘1’. (**b**) Bit bar line voltage (*V*_QB_) for various |*V*_BS_| with transition from ‘0’ to ‘1’. (**c**) *V*_Q_ for various |*V*_BS_| with stay from ‘1’ to ‘1’. (**d**) *V*_QB_ for various |*V*_BS_| with stay from ‘1’ to ‘1’.

**Figure 3:**
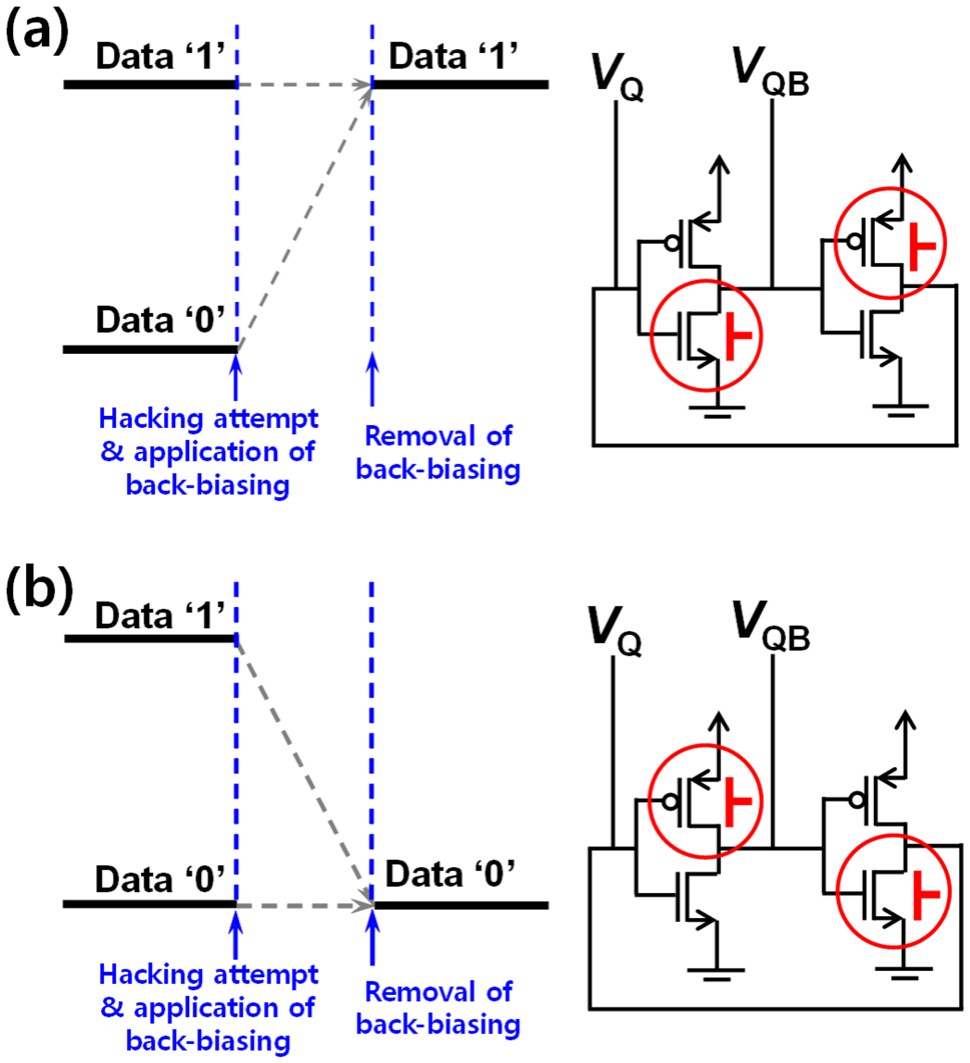
Simplified data diagram with the asymmetric forward back-biasing scheme for permanent erasing. (**a**) All the data are reset to ‘1’. (**b**) All the data are reset to ‘0’. Whether all the data were reset to ‘1’ or ‘0’ is determined according to the asymmetric forward back-biasing scheme.

Figure 2 shows the results of permanent erasing by use of forward back-biasing at room temperature. Figure 2a and b show the bit line voltage (*V*_Q_) and bit bar line voltage (*V*_QB_) when ‘0’ was stored initially. Note that *V*_Q_ and *V*_QB_ have contrasted voltage levels for the same data state. For example, *V*_Q_ and *V*_QB_ have 0 V and 1 V for the data state ‘0’, respectively. *V*_Q_ and *V*_QB_ are changed by the applied forward back-bias (|*V*_BS_|). When positive *V*_BS_ was forwardly applied to the NMOS of the left inverter and negative *V*_BS_ was forwardly applied to the PMOS of the right inverter with the same magnitude of more than 0.9 V (Fig. 1c), the initial *V*_Q_ of 0 V was changed to 1 V and the initial *V*_QB_ of 1 V was changed to 0 V. Therefore, the initial ‘0’ was pulled up to a final ‘1’. Figure 2c and d show *V*_Q_ and *V*_QB_ when ‘1’ was stored initially. With the same forward back-biasing, as shown in Fig. 2a and b, *V*_Q_ was maintained as 1 V and *V*_QB_ also remained at 0 V. Hence the initial ‘1’ was sustained as final ‘1’. As a consequence, stored data were reset to ‘1’ *en bloc*, regardless of the initial data state. Figure 3a and b show simplified data diagrams and corresponding circuits for the permanent erasing. The erased states to ‘1’ were sustained even after the back-biasing was removed, as shown in Fig. 3a. In contrast, ‘0’ and ‘1’ were reset to ‘0’ *en bloc*, as another permanent erasing when the forward back-bias was applied to the PMOS of the left inverter and the NMOS of the right inverter, as shown in Fig. 3b.

**Figure 4:**
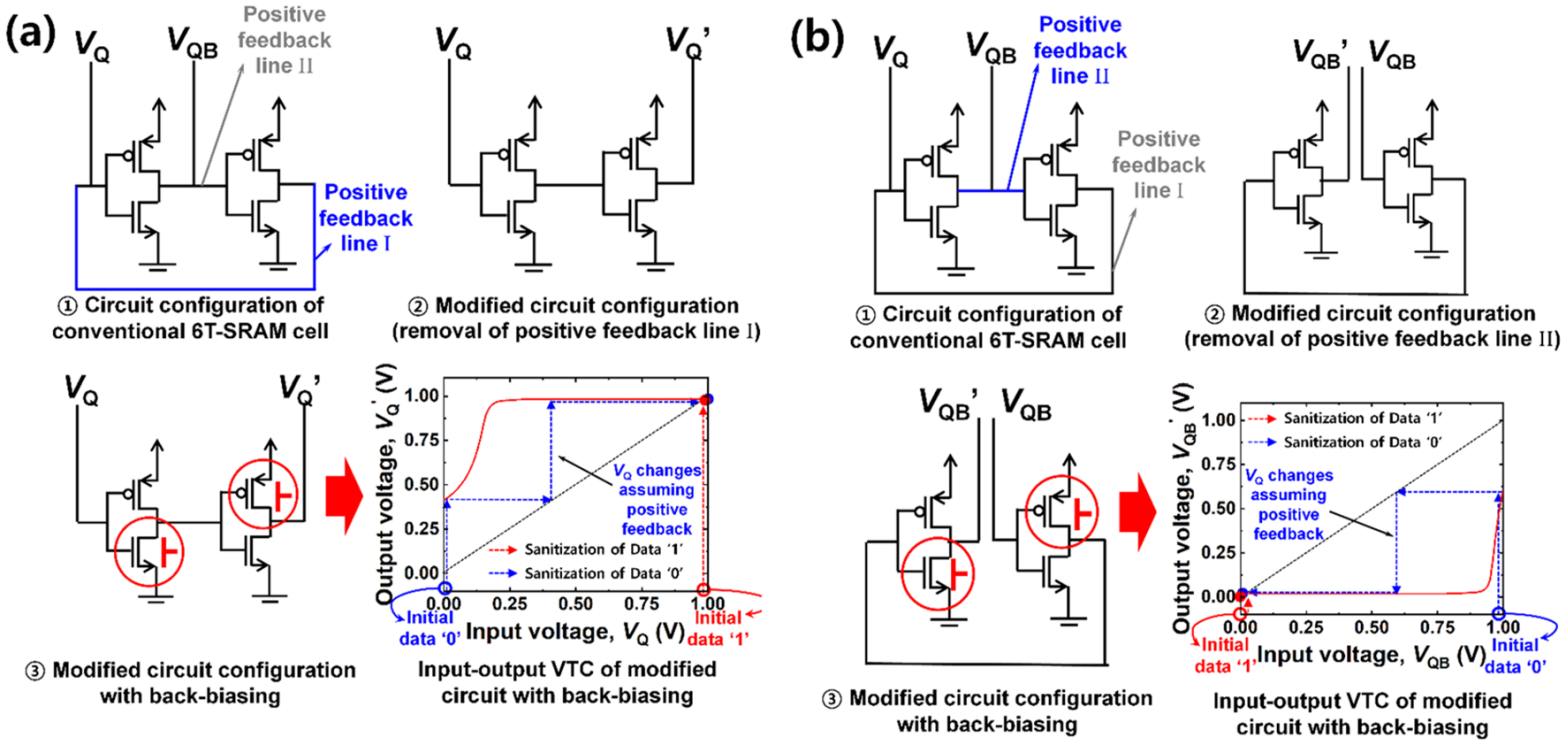
Modified configuration of a 6 T-SRAM cell with its corresponding input–output voltage transfer curve (VTC) by asymmetric forward back-biasing for permanent erasing to state ‘1’. (**a**) Circuit diagrams with two conventional positive feedback lines (I & II) and without positive feedback line I and the VTC in terms of bit line voltage (*V*_Q_) to show distorted latch in SRAM. The initial *V*_Q_ of 0 V (data ‘0’) and 1 V (data ‘1’) were changed to 1 V (data ‘1’). (**b**) Circuit diagrams with two conventional positive feedback lines (I & II) and without positive feedback line II and the VTC in terms of bit bar line voltage (*V*_QB_) to show distorted latch in SRAM. The initial *V*_QB_ values of 1 V (data ‘0’) and 0 V (data ‘1’) were changed to 0 V (data ‘1’). All the data are reset to ‘1’.

A body terminal in a MOSFET can serve as a secondary gate (pseudo-gate), while an actual gate terminal can operate as a primary gate. Herein when an initially off-state MOSFET in an inverter was turned on by the applied back-bias, the latch state in the cross-coupled inverters was notably distorted. Figure 4 explains how the latch state is distorted and thereby data are permanently erased to state ‘1’. The configuration of a 6 T-SRAM cell was intentionally modified to analyze the distortion of the latch state. Figure 4a and b show the modified circuit configuration and its input–output voltage transfer curve (VTC) with the back-biasing. Two positive feedback lines, I and II, are separately removed from the conventional 6 T-SRAM cell. Thereafter, forward back-bias was applied to the modified cell in order to extract the distorted VTC of *V*_Q_ and *V*_QB_. *V*_Q_’ and *V*_QB_’ were defined as the output voltage through two inverters that receive input *V*_Q_ and *V*_QB_, respectively. In the case where positive feedback line I is removed, shown in Fig. 4a, the blue rectilinear dashed-line in the VTC graph shows how *V*_Q_ is changed, when data ‘0’ was initially stored in the modified VTC graph. The initial *V*_Q_ of 0 V (data ‘0’) was pulled up to 1 V (data ‘1’) by the removal of positive feedback line I. The red vertical dashed-line in the VTC graph shows how *V*_Q_ is changed, when data ‘1’ was initially stored. The initial *V*_Q_ of 1 V (data ‘1’) was maintained by the removal of positive feedback line I. Therefore, the permanent data erasing can proceed by making both data ‘0’ and ‘1’ into ‘1’. In the case of removing positive feedback line II, shown in Fig. 4b, the blue rectilinear dashed-line in the VTC graph shows how *V*_QB_ is changed, when data ‘0’ was initially stored. The initial *V*_QB_ of 1 V (data ‘0’) was pulled down to 0 V (data ‘1’) by the removal of positive feedback line II. The red vertical dashed-line in the VTC graph shows how *V*_QB_ is changed, when data ‘1’ was initially stored. The initial *V*_QB_ of 0 V (data ‘1’) was maintained by removing positive feedback line II. Therefore, the permanent data erasing can also proceed by making both data ‘0’ and ‘1’ into ‘1’. While the other permanent data erasing shown in Fig. 3b is not explained, it similarly works as described above. In this case, all the data of ‘0’ and ‘1’ can be reset to ‘0’.

**Figure 5:**
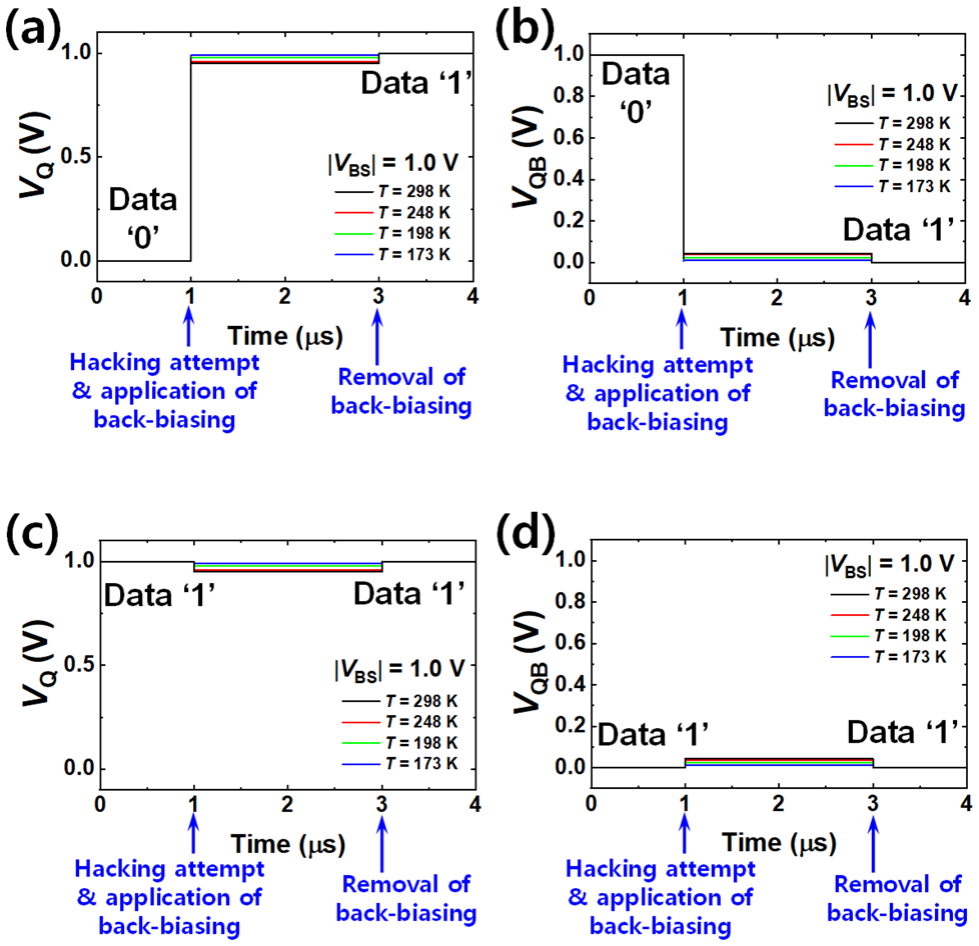
Temperature-invariant data distortion by permanent erasing with |*V*_BS_| of 1 V. (**a**) and (**b**) are for when the initial data was ‘0’. (**c**) and (d) are for when the initial data was ‘1’. (**a**) Distorted *V*_Q_ from ‘0’ to ‘1’ for various *T*. (b) Distorted *V*_QB_ from ‘0’ to ‘1’ for various *T*. Data ‘0’ was changed to data ‘1’ regardless of *T*. (**c**) Distorted *V*_Q_ with stay of ‘1’ for various *T*. (**d**) Distorted *V*_QB_ with stay of ‘1’ for various *T*. Data ‘1’ was maintained regardless of *T*. All the data were reset to ‘1’-state after the permanent erasing even at low *T* against the cold boot attack.

In order to resist a cold boot attack, the proposed data erasing by forward back-biasing must be available in a low temperature environment. Therefore, it was confirmed that permanent data erasing was achievable at low temperatures down to 173 K^6,7^. The abovementioned cryogenic (CRYO) model was used for accurate physics-based low temperature simulations. Figure 5a and b show *V*_Q_ and *V*_QB_ when data ‘0’ was initially stored, and Fig. 5c and d exhibit *V*_Q_ and *V*_QB_ when data ‘1’ was initially stored for various temperatures ranging from 173 to 298 K. The data was reset to ‘1’ by the forward back-biasing even at *T* of 173 K. This is because *ΔT* (= *T*_room_—*T*_low_) of 125 K (= 298 K – 173 K) makes a small positive *ΔV*_T_ of 0.12 V and *ΔV*_BS_ (= *V*_BS,GND_—*V*_BS,FWD_) of -1 V (= 0 V – 1 V) induces a large negative *ΔV*_T_ of 0.66 V in an NMOS; *i*.*e*., the *V*_T_ change by the forward back-biasing overwhelms the *V*_T_ change by the temperature.

Supplementary Fig. S1a shows how the erasing time varies according to the load capacitance (*C*_L_) connected to each inverter in a SRAM cell. It corresponds to bit-line capacitance, which tends to be decreased as a technology node is advanced. Referring to^16^, *C*_L_ is 117 fF at a 70 nm technology node. The erasing time is increased by prolonged *RC* delay, as *C*_L_ is increased. Therefore, the erasing time can be increased with larger *C*_L_ in larger array architecture. However, as the technology node advances, fast data sanitization is possible due to reduced *C*_L_. For example, the ultra-fast data sanitization within 5 ns is feasible for *C*_L_ of 1 pF, which is larger than nominal *C*_L_ below 100 fF^18^ at the 32 nm node. It is worth noting that the erasing time can be further shortened by increment of |*V*_BS_|. Supplementary Fig. S1b shows the erasing time influenced by *C*_L_ for various temperatures. Ultra-fast erasing within 5 ns is also achievable even at 173 K (Supplementary Fig. S2a). This implies that the proposed bask-biasing scheme can resist the cold boot attack.

Meanwhile, if |*V*_BS_| is larger than the built-in potential (~ 0.8 V) of the p–n junction at the source and drain, a forward junction current (*I*_j,FWD_) is flown^18^. From the simulation, *I*_j,FWD_ values of 0.29 mA and 2.13 mA were flown for |*V*_BS_| of 0.9 V and 1 V, respectively. Accordingly, power consumption for the permanent erasing was extracted as 0.58 mW and 4.26 mW for the |*V*_BS_| of 0.9 V and 1 V, respectively. However, the energy consumption for the permanent erasing could be reduced to an order of pJ. This is because shorter time than 5 ns is sufficient to delete the data in the ultra-fast sanitization.

### Temporary erasing

**Figure 6:**
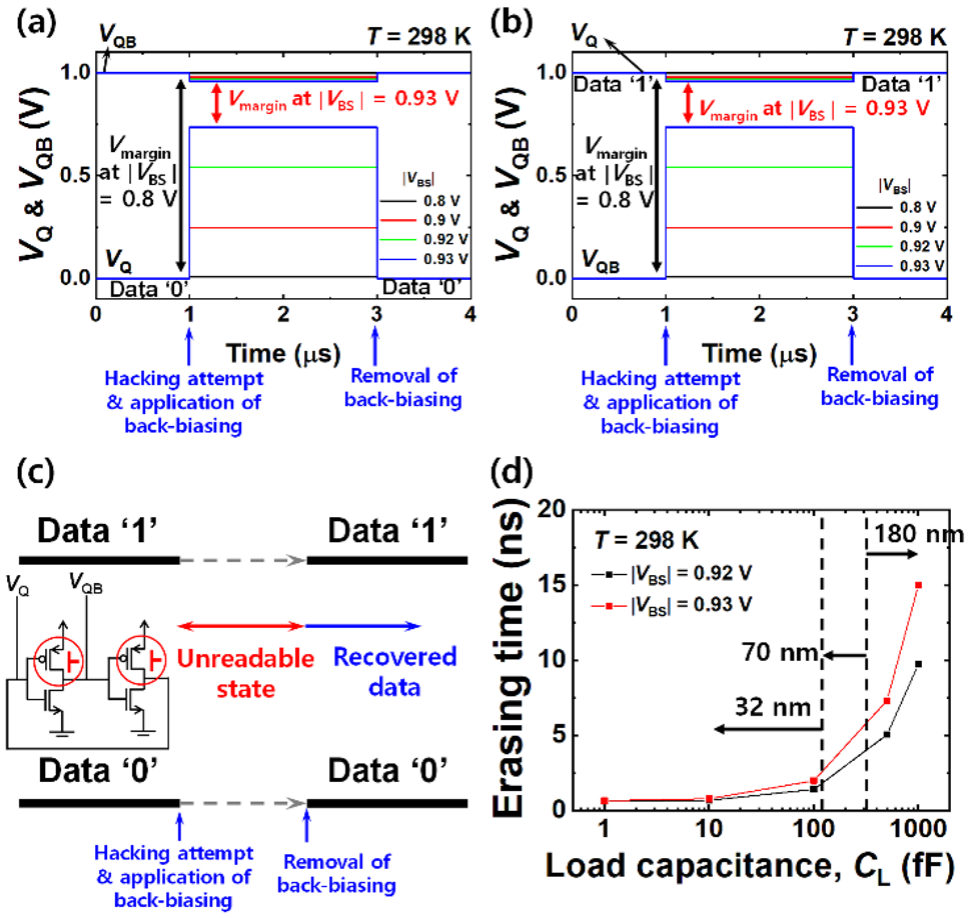
(Temporary erasing) Bit line voltage (*V*_Q_) and bit bar line voltage (*V*_QB_) depending on the |*V*_BS_| at *T* = 298 K, when the initial data was (**a**) ‘0’ and (**b**) ‘1’. *V*_margin_ reduction was achieved by applied |*V*_BS_|. (**c**) Simplified data diagram with the asymmetric forward back-biasing scheme for temporary erasing. (**d**) Erasing time versus load capacitances for various technology nodes: 32 nm, 70 nm and 180 nm.

Figure 6 shows the results of the temporary erasing with forward back-biasing at room temperature. Figure 6a and b show *V*_Q_ and *V*_QB_ when ‘0’ and ‘1’ were respectively stored. They were modulated by the applied *V*_BS_. When negative *V*_BS_ (*i*.*e*., forward back-biasing) applied to both PFETs in two inverters was increased, the voltage margin (*V*_margin_) between *V*_Q_ and *V*_QB_ was narrowed. Note that the minimal *V*_margin_ for normal reading operation in SRAM is 0.25 V to distinguish ‘0’ and ‘1’^19^. From the simulation, the corresponding value was 0.22 V at a |*V*_BS_| of 0.93 V. Thus, the latched data state in the SRAM cell is seriously distorted to the point of being illegible because its *V*_margin_ is smaller than 0.25 V. Figure 6c shows a simplified data diagram of the temporary erasing. When *V*_margin_ is small enough to be indistinguishable, a hacker cannot read the data. On the other hand, unreadable data by temporary erasing can be promptly recovered to their original states by removing the back-bias after the threat of the attack has disappeared. Figure 6d shows the erasing time of the temporary erasing, which was affected by *C*_L_. The data sanitization could be accomplished within 15 ns, which is sufficiently fast. The slight difference between temporary erasing time and permanent erasing time is attributed to the different back-bias scheme. Recall that symmetric back-biasing was applied for the temporary erasing and asymmetric back-biasing was applied for the permanent erasing. This results in a dissimilar *RC* delay affecting the time to distort the stored data in SRAM.

**Figure 7:**
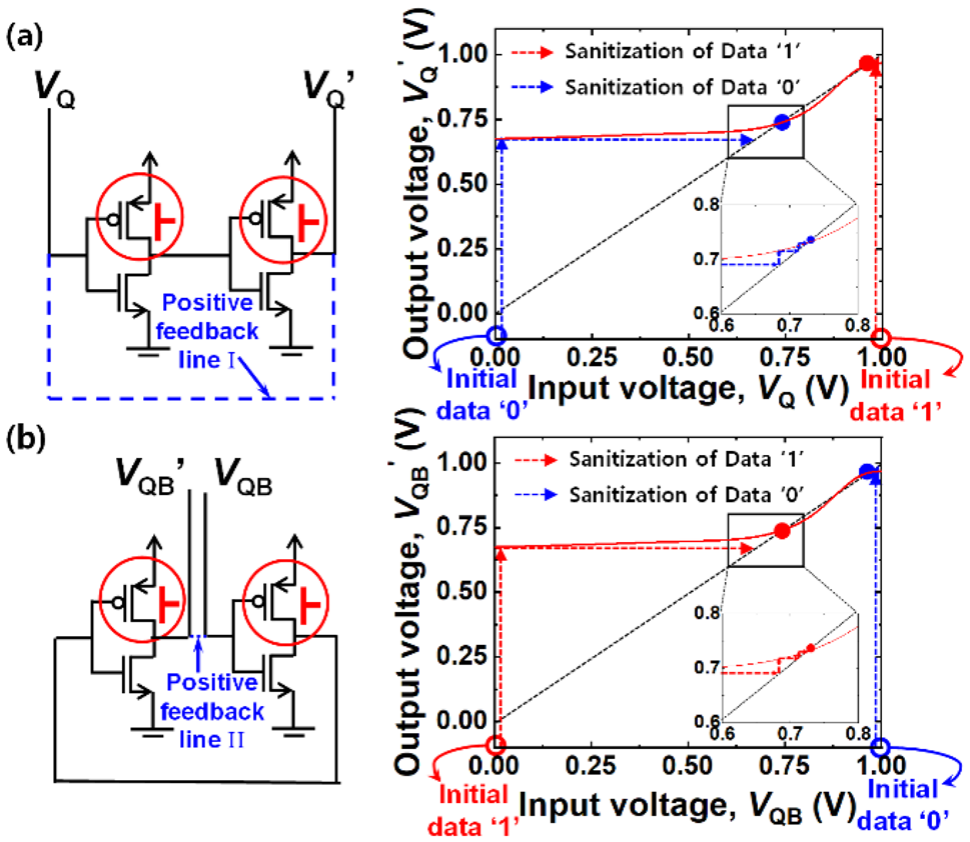
Modified configuration of a 6 T-SRAM cell with its corresponding input–output voltage transfer curve (VTC) by symmetric forward back-biasing for temporary erasing. (**a**) Circuit diagram without positive feedback line I and its VTC in terms of bit line voltage (*V*_Q_) to show partially distorted latch. The initial *V*_Q_ values of 0 V (data ‘0’) and 1 V (data ‘1’) were changed to 0.73 V and 0.95 V, respectively. (**b**) Circuit diagram without positive feedback line II and its VTC in terms of bit bar line voltage (*V*_QB_) to show partially distorted latch. The initial *V*_QB_ values of 1 V (data ‘0’) and 0 V (data ‘1’) were changed to 0.95 V and 0.73 V, respectively. *V*_margin_ of 0.22 V is achieved for both temporary erasing of data ‘0’ and ‘1’.

Like the permanent erasing by the aforementioned asymmetric back-biasing, the temporary erasing can also partially disturb the latch state by symmetric back-biasing. However, the level of the disturbance in the temporary erasing is small compared with that in the case of permanent erasing. Figure 7 shows how much the latch state is disturbed by the temporary erasing and thereby data become temporarily unreadable. Figure 7a and b show the modified circuit configuration and its input–output VTC with the back-biasing. Being done at Fig. 4, two positive feedback lines I and II were also individually removed from the conventional 6 T-SRAM cell, as depicted in each circuit diagram of Fig. 7. Thereafter, forward back-bias was applied to the modified SRAM cell in order to extract the distorted VTC of *V*_Q_ and *V*_QB_. *V*_Q_’ and *V*_QB_’ were defined as the output voltage through the modified cross-coupled inverters that receive input *V*_Q_ and *V*_QB_, respectively. Referring to the VTC graph from Fig. 7a, the blue rectilinear dashed-line shows how much *V*_Q_ is changed, when data ‘0’ was initially stored. The initial *V*_Q_ of 0 V (data ‘0’) was pulled up to 0.73 V by removing positive feedback line I. The red vertical dashed-line in the VTC graph shows how *V*_Q_ is changed, when data ‘1’ was initially stored. The initial *V*_Q_ of 1 V (data ‘1’) was pulled down to 0.95 V by the removed positive feedback line I. Likewise, referring to the VTC graph in Fig. 7b, the blue vertical dashed-line shows how much *V*_QB_ is changed, when data ‘0’ was initially stored. The initial *V*_QB_ of 1 V (data ‘0’) was pulled down to 0.95 V by removal of positive feedback line II. The red rectilinear dashed-line in the VTC graph shows how much *V*_QB_ is changed, when data ‘1’ was initially stored. The initial *V*_QB_ of 0 V (data ‘1’) was pulled up to 0.73 V by removal of positive feedback line II. Therefore, *V*_margin_ becomes 0.22 V (= 0.95—0.73 V) for both temporary erasing of data ‘0’ and ‘1’. Herein *V*_Q_ of 0.73 V and *V*_QB_ of 0.95 V (or vice versa) are too ambiguous to be classified as one of a binary data state: 0 V and 1 V. Moreover, the temporarily disturbed *V*_Q_ and *V*_QB_ by the abovementioned pulled-up or pulled-down operation could be recovered to their initial states by removal of the back-biasing, because the final order of *V*_Q_ > *V*_QB_ or *V*_Q_ < *V*_QB_ inherited from their initial order was not reversed.

**Figure 8:**
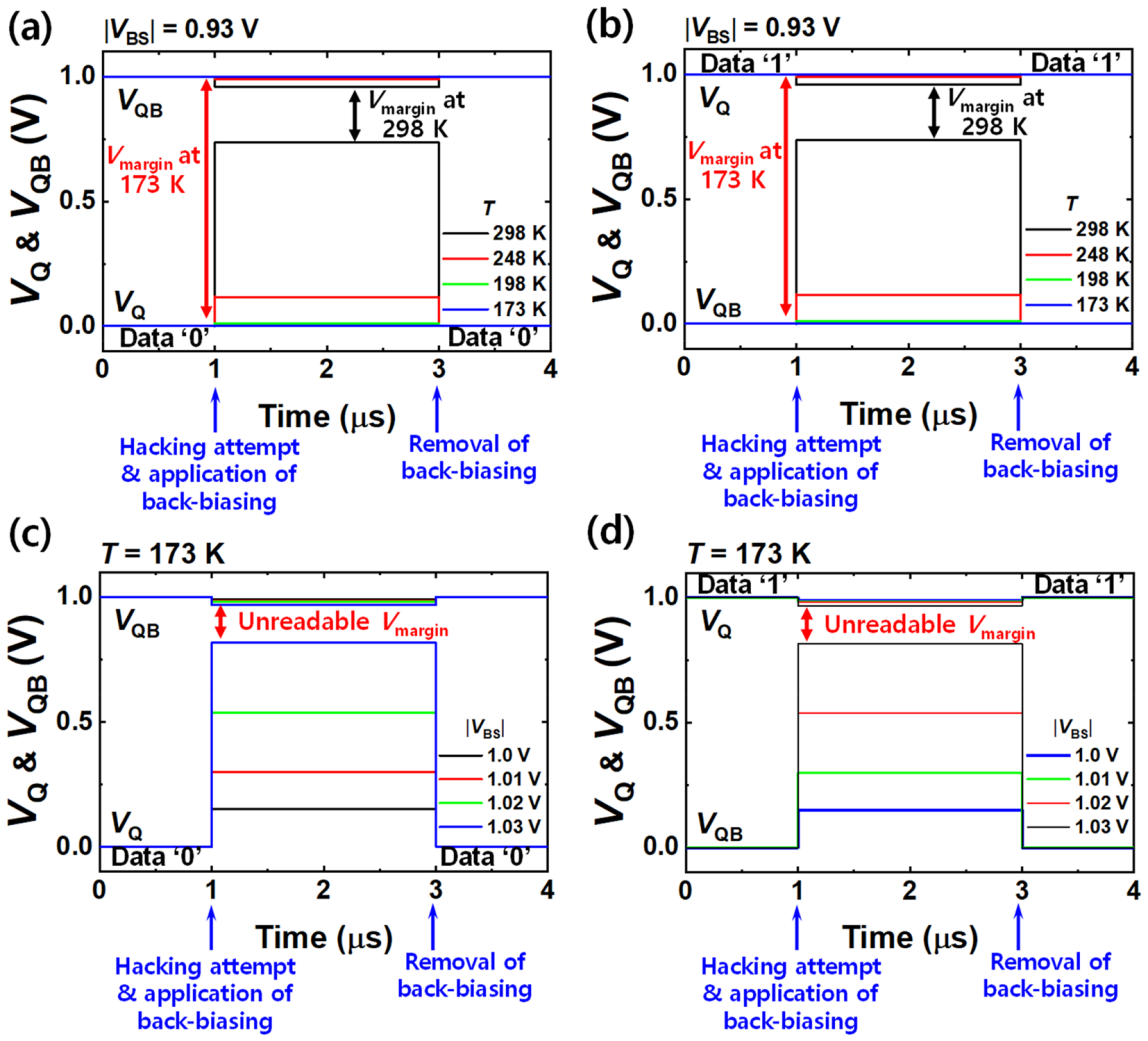
Temperature-variant partial data distortion by temporary erasing for various |*V*_BS_|. (**a**) *V*_Q_ and *V*_QB_ for various temperatures at |*V*_BS_| of 0.93 V, when the initial data was ‘0’. (**b**) *V*_Q_ and *V*_QB_ for various temperatures at |*V*_BS_| of 0.93 V, when the initial data was ‘1’. At 173 K, widened *V*_margin_ (close to 1 V) under |*V*_BS_| of 0.93 V is vulnerable to a hacking attempt. (**c**) *V*_Q_ and *V*_QB_ for various |*V*_BS_| at 173 K, when the initial data was ‘0’. (d) *V*_Q_ and *V*_QB_ for various |*V*_BS_| at 173 K, when the initial data was ‘1’. Temporary erasing is possible even at low temperature by further increment of |*V*_BS_|.

The temporary erasing at low temperature was also investigated with simulations to confirm whether it can resist the cold boot attack. Figure 8a and b show *V*_Q_ and *V*_QB_ depending on the temperature, when a |*V*_BS_| of 0.93 V was applied. This is the condition where the temporary erasing worked at room temperature. As the temperature was decreased to 173 K, *V*_margin_ was notably widened to nearly 1 V again. Thus, data sanitization by the temporary erasing could not be accomplished. This vulnerability to low temperature can be mitigated by increasing |*V*_BS_|. Figure 8c and d show *V*_Q_ and *V*_QB_ as a function of |*V*_BS_| at 173 K. As |*V*_BS_| was increased, *V*_margin_ narrowed. Temporary erasing time within 5 ns was also achievable even at 173 K (Supplementary Fig. S2b). Conclusively, it is confirmed that the temporary erasing as well as the permanent erasing can resist the cold boot attack by the forward back-biasing.

It should be noted that the proposing data sanitization requires a new layout scheme in order to separately apply the back-bias. Supplementary Fig. S3a shows a conventional layout of high-density 6 T-SRAM. According to the conventional 6 T-SRAM layout, two pull-up (PU) PFETs share the same N-type well so that it is impossible to provide different back-bias for each PU PFET. In order to provide back-bias to only one of the PU PFET, the layout innovation is required. Supplementary Fig. S3b shows a possible layout innovation to realize individual back-biasing by using two N-type wells, which are separated by a slim P-type well. In addition, as shown in Supplementary Fig. S3a, pull-down (PD) NFET and pass-gate (PG) NFET share the same P-type well. Therefore, if the back-bias is applied to the P-type well, the back-bias will influence on the PG and PD NFET together because they are located in the same well. One of the solutions is to apply negative voltage to the GND node rather than to apply positive voltage to the P-type well for the back-biasing. More specifically, if the GND is divided to GND1 and GND2 as shown in the Supplementary Fig. S3b, two PD NFETs can be biased individually. The cross-sections of conventional layout of high-density 6 T-SRAM and possible layout innovation to realize proposed data sanitization scheme are shown in Supplementary Fig. S4. In a layout point of view, a concern to the proposed SRAM cell to allow the back-biasing is a slim p-type well that has a width in a range of 80 nm, which is close to a lithographic limit in a 32 nm technology node. Supplementary Fig. S5 compares a footprint area between a conventional layout and the proposed layout in a 6 T-SRAM cell. According to^21^, the area of SRAM cell in the 32 nm technology is 0.74 μm × 0.27 μm. Therefore, the area of the proposed SRAM cell is expected to be increased to 10.8% compared with the conventional one owing to an additional 80 nm well width. The well-proximity effect (WPE) may provoke an *V*_T_ shift in a proposed cell transistor. But, it is simply compensated for an invariant noise margin by employment of channel *V*_T_ implantation, which has commonly used in CMOS fabrication. Table 1 shows the area comparison among various implementations for SRAM sanitization^22–24^. Each implementation has a different cell size according to a sanitization type. Note that the proposed approach has the smallest number of transistors with the reduced normalized cell size.

**Table 1:** Comparison of various implementations for SRAM sanitization.

	Cell type	Sanitization type	Number of transistors	Normalized cell size
–	Convential 6 T-SRAM	–	6	1
^21^	8 T-SRAM	Power-cut	8	1.81
^22^	8 T-SRAM	Feedback-cut	8	1.41
^23^	7 T-SRAM	Voltage equalizing	7	1.78
This work	6 T-SRAM	Back-biasing	6	1.1

To generate the back-bias, a controller should detect whether the SRAM is under attack. Supplementary Fig. S6 shows a strategy to detect the cold boot attack, which will be served as a trigger to enable the data sanitization. As soon as the cold boot attack is attempted by lowering temperature of a SRAM chip to a cryogenic level, such threatening should be detected prior to actual hacking. Herein, a CMOS temperature-to-pulse generator can be used to sense the lowering of ambient temperature, as shown in Supplementary Fig. S7. A delay time is induced between two transmission lines when the ambient temperature is changed, and a XOR gate generates pulses according to the mismatch in the two lines^25^. By connecting the output of the CMOS temperature-to-pulse generator to a back-bias terminal, the data in the SRAM cell can be instantly erased when a rapid cooling is attempted by liquid nitrogen. In this way, the back-bias for SRAM data sanitization can be applied automatically.

## Discussion

For a security system, ultra-fast data sanitization for SRAM was demonstrated with forward back-biasing, which did not require any extra circuit. The simulation study confirmed that this strategy could resist the cold boot attack. The latch states in SRAM were distorted by the forward back-biasing in order to reset data or make data unreadable against hacking. The level of the distortion was modulated by various back-biasing schemes. Symmetric back-biasing to two PMOS supported recoverable temporary erasing. Furthermore, asymmetric back-biasing to the PMOS in one inverter and to the NMOS in the other inverter facilitated irrecoverable permanent erasing. According to the level of the hacking threat, either permanent erasing or temporary erasing can be chosen by an end user. Based on the physics-based ATLAS device simulations, the possibility of data sanitization at low temperature down to 173 K in order to resist a cold boot attack was confirmed. In the meanwhile, the direct demonstration of the sanitization with a fully fabricated SRAM chip is planned for the justification of actual security hardware in future.

## Supplementary Information

Supplementary Information.

## Data Availability

*Scientific Reports* requires the inclusion of a data availability statement with all submitted manuscripts, as this journal requires authors to make available materials, data, and associated protocols to readers.
