# Supplementary material for: Ultra-fast data sanitization of SRAM by back-biasing to resist a cold boot attack
Source: Sci Rep. 2022 Jan 7;12:35. doi: 10.1038/s41598-021-03994-2 (PMC8742074; doi:10.1038/s41598-021-03994-2)
Supplement: Supplementary file 1 — Supplementary Information. [file 41598_2021_3994_MOESM1_ESM.docx]

**Supplementary Information**

Ultra-fast Data Sanitization of SRAM by Back-biasing to Resist a Cold Boot Attack

Seong-Joo Han^1†^, Joon-Kyu Han^1†^, Gyeong-Jun Yun^1^, Mun-Woo Lee^1^, Ji-Man Yu^1^, and Yang-Kyu Choi^1,a)^

^1^ School of Electrical Engineering, Korea Advanced Institute of Science and Technology, (KAIST) 291 Daehak-ro, Yuseong-gu, Daejeon 34141, Republic of Korea
^*^ykchoi@ee.kaist.ac.kr

^⸸^these authors contributed equally to this work

**Erasing time versus load capacitance for various technology nodes**

**Figure S1.** Erasing time versus load capacitance for various technology nodes: 32 nm, 70 nm and 180 nm. **(a)** Erasing time as a function of load capacitance (*C*_L_) for various |*V*_BS_| at room temperature (*T* = 298 K). **(b)** Erasing time as a function of *C*_L_ for various *T* at |*V*_BS_| = 1.0 V.

**Erasing time depending on load capacitance**

**Figure S2.** Capacitance dependent erasing for **(a)** permanent and **(b)** temporary erasing at 298 K and 173 K.

**Layout innovation for back-bias utilized 6T-SRAM**

**Figure S3. (a)** Conventional layout of high-density 6T-SRAM. **(b)** Proposed layout for data sanitization by back-biasing.

**Cross-section of high-density 6T-SRAM layout**

**Figure S4.** Cross-section of conventional layout for high-density 6T-SRAM and proposed layout for data sanitization by back-biasing. P-type channel-stop implantation with high dose can be utilized for compact twin N-wells.

**Figure S5.** Area comparison between conventional layout and proposed layout in the high-density 6T-SRAM cell.

**Strategies of detecting cold boot attack**

**Figure S6.** Strategy to sense a cold boot attack and trigger the data sanitization.

**Figure S7.** Feasible concept of a CMOS temperature-to-pulse generator.
